# Yeast central nervous system infection in a critically ill patient: a case report

**DOI:** 10.1186/1752-1947-8-253

**Published:** 2014-07-15

**Authors:** Frantzeska Frantzeskaki, Chryssi Diakaki, Michalis Rizos, Maria Theodorakopoulou, Panagiotis Papadopoulos, Anastasia Antonopoulou, Nikitas Nikitas, Michail Lignos, Elias Brountzos, Aristea Velegraki, Elisabeth Paramythiotou, John Panagyotides, Apostolos Armaganidis, George Dimopoulos

**Affiliations:** 1 2nd Department of Critical Care, University Hospital ATTIKON, Athens, Greece; 2 Department of Radiology, University Hospital ATTIKON, Athens, Greece; 3 Microbiology department, Mycology research laboratory, Medical school, National and Capodistrian University of Athens, Athens, Greece; 4 2nd Pathology Department, University Hospital ATTIKON, Athens, Greece

**Keywords:** Emerging fungal infections, ICU patients, Treatment

## Abstract

**Introduction:**

Invasive fungal infections are alarmingly common in intensive care unit patients; invasive fungal infections are associated with increased morbidity and mortality. Risk factors are the increased use of indwelling central venous catheters, the use of broad spectrum antibiotics, parenteral nutrition, renal replacement therapy and immunosuppression. Diagnosis of these infections might be complicated, requiring tissue cultures. In addition, therapy of invasive fungal infections might be difficult, given the rising resistance of fungi to antifungal agents.

**Case presentation:**

We describe the case of a 28-year-old Greek man with yeast central nervous system infection.

**Conclusions:**

Difficult-to-treat fungal infections may complicate the clinical course of critically ill patients and render their prognosis unfavorable. This report presents a case that was rare and difficult to treat, along with a thorough review of the investigation and treatment of these kinds of fungal infections in critically ill patients.

## Introduction

Invasive fungal infections are increasingly common in intensive care unit (ICU) patients and are associated with prolonged hospitalization duration and increased mortality [1]. The worldwide Extended Prevalence of Infection in Intensive Care study conducted in 2007 showed that almost 20% of all isolated pathogens in ICU patients were fungi, with *Candida* spp*.* ranking fourth after *Staphylococcus* spp*.*, *Pseudomonas* spp. and *Escherichia coli. Candida* spp. were the most frequently isolated yeasts, responsible for almost 88% of fungal infections. Of interest, there is an increasing trend of fungal infections caused by non-*albicans Candida* species, relatively resistant to commonly used antifungal agents [2,3]. The cited attributable mortality for *Candida* infections varies from 5% to 71% [4]. The increased incidence of fungal infections in ICU patients may be attributed to a variety of reasons such as the increasing incidence of immunocompromised patients requiring ICU admission, the ageing population of ICU patients, and the large number of invasive medical practices required in ICUs [5]. This report presents a difficult to treat central nervous system (CNS) fungal infection in a medical-surgical ICU (MSICU) of a tertiary hospital.

## Case presentation

**Figure 1:**
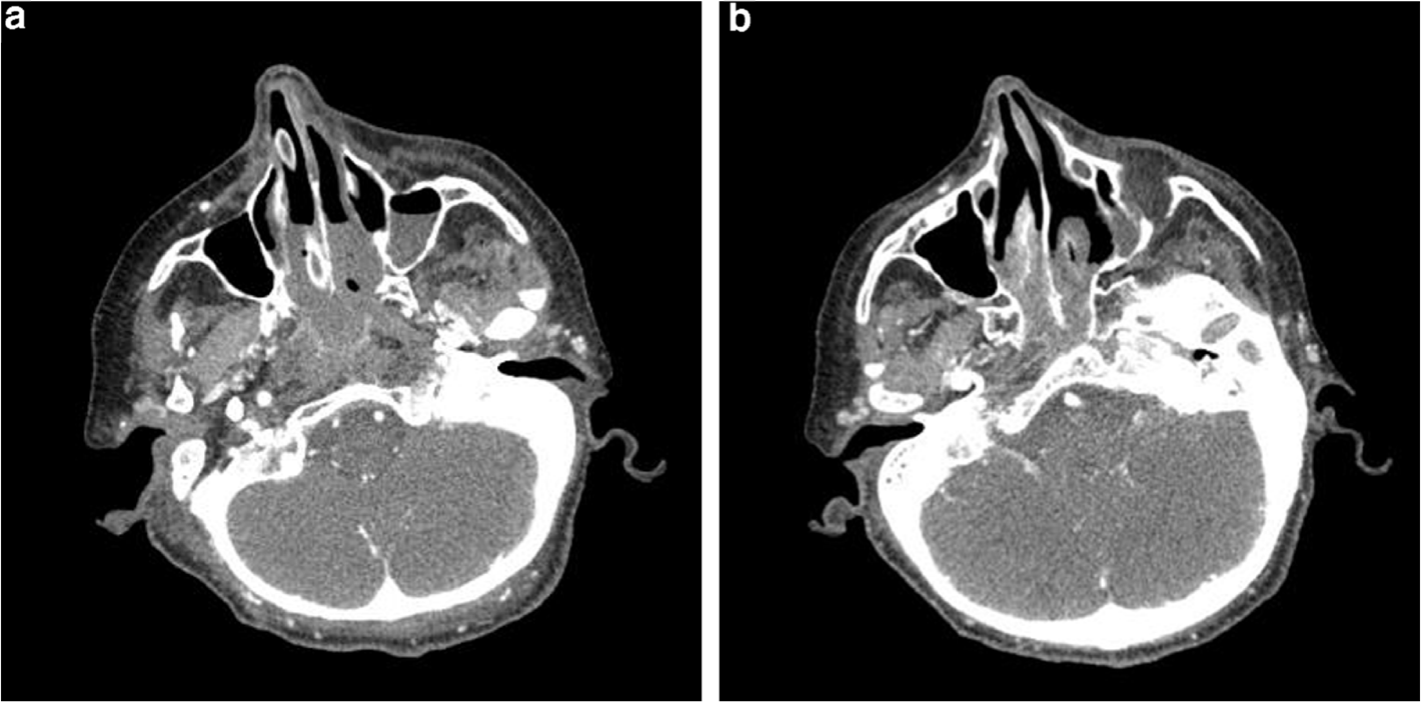
(a and b) A brain computed tomography scan showing blurred bony structures of the floor of the mouth and the tongue, consolidation of left maxillary sinus and accompanying invasion of the maxillary bone.

**Figure 2:**
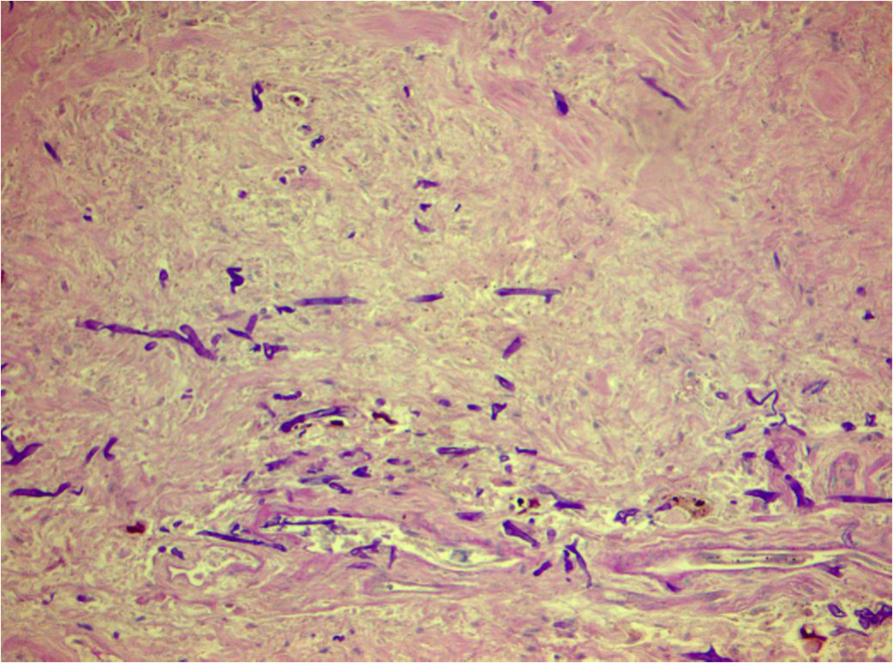
**Gram stain (original magnification: ×1000).** Yeast cells found after examination of multiple cerebrospinal fluid smears.

A 28-year-old Greek man was admitted to the neurological department of a tertiary hospital with drop of the right corner of his mouth, left eyelid ptosis, bilateral visual field defects, diplopia, headache, fever and dizziness. He had been diagnosed with Hodgkin’s lymphoma 18 months earlier and had achieved complete remission after eight courses of chemotherapy. Four months prior to this admission recurrence of the disease was diagnosed, and he underwent new courses of salvage treatment with etoposide, methylprednisolone, high-dose cytarabine and cisplatin (ESHAP). The last course was performed a month before the present admission and a follow-up positron emission tomography scan showed minimal residual disease. He was not receiving any antifungal prophylaxis. On his admission, a brain contrast-enhanced computed tomography (CT) scan was normal and a lumbar puncture yielded cerebrospinal fluid (CSF) with 175 leukocytes/mm^3^ (lymphocytes 98%), an elevated protein level of 128mg/dL and a reduced glucose level of 35mg/dL (120mg/dL in serum). Gram stain, cultures, *Cryptococcus* antigen and polymerase chain reaction (PCR) for herpes viruses were negative. Blood cultures were negative. Magnetic resonance imaging of his brain disclosed high signal intensity of fast fluid-attenuated inversion recovery, involving periventricular and subcortical gray matter of bilateral brain hemispheres, hippocampus, internal capsule bilaterally, thalami, pons, cerebral peduncles, substantia nigra of midbrain, middle and inferior cerebellar peduncles, and cervical spinal cord, without hemorrhage nor restricted diffusion pattern (Figures 1a and 1b). After administration of a paramagnetic substance, leptomeningeal contrast enhancement was noticed, and the above lesions accentuated. A brain biopsy was performed and the pathologic examination of dura mater specimens showed yeast cells (periodic acid–Schiff histochemical stain). A panfungal PCR assay was arranged for brain tissue specimens. A second lumbar puncture was performed: CSF cell counts showed 100 leukocytes/mm^3^ (lymphocytes 85%), glucose 40mg/dL (120mg/dL in serum) and protein level 100mg/dL. Gram stain, India ink preparation and cultures remained negative. However, yeast cells were observed on a second Gram stain examination of CSF (Figure 2). A diagnosis of “yeast” CNS infection was established and he was empirically treated with liposomal amphotericin B (450mg once a day intravenous) and flucytosine (100mg/kg/day divided into four oral doses). Five days later his level of consciousness deteriorated and tracheal intubation was performed because of impending coma. He was admitted to ICU and a new brain CT was performed showing multiple ring-like enhanced lesions with peripheral edema affecting the gray matter of l hemispheres bilaterally. A week later, while he was still in a comatose condition, he suddenly presented dilatation of pupils, predominately of his left one, with no reaction to light. A new brain CT showed diffuse brain edema affecting mainly his posterior cranial fossa, indicating tentorial herniation. Despite the administered osmotherapy with dexamethasone and mannitol, he developed cardiac asystole on the same day and died. An in-house real-time panfungal PCR assay (LightCycler, Roche®) performed following automated deoxyribonucleic acid (DNA) extraction (Maxwell 16®, Promega) from fresh brain tissue specimens was positive for yeast DNA.

## Discussion

This report describes a yeast-related CNS infection of a young immunocompromised man. Yeasts are pathogenic in patients with hematological neoplasms (as in our patient), solid neoplasms, acquired immunodeficiency syndrome and transplantation of bone marrow or solid organs. In our case, blood cultures were negative, as might happen in 50% of CNS fungal infections. However, diagnosis was confirmed by microscopy. In our patient the PCR assay of brain tissue was yeast positive. Amphotericin B is the drug of choice, in the absence of positive cultures and hence inability to perform susceptibility testing [6,7]. Despite treatment, he died. More studies are required in order to clarify the exact physiological mechanism of yeast CNS infection and the appropriate treatment. The entity is uncommon but affects predominantly neonates, neurosurgical patients and immunosuppressed patients. It has been described in patients with granulomatous disease, myeloperoxidase deficiency, severe combined immunodeficiency, human immunodeficiency virus infection, organ transplantation and lymphoma [8]. Fungemia might precede CNS infection, while direct inoculation of the fungus may occur, for example after placement of CNS prostheses [8]. However, in our case, blood cultures were negative, as might happen in 50% of fungemias. *Candida albicans* accounts for 70% to 100% of all *Candida* CNS infection cases [9]. Table 1 depicts the previously reported cases of *Candida* CNS infections. These may include cerebral microabscesses, manifesting as diffuse encephalopathy, or cerebral abscesses with focal neurologic signs, and meningitis, as in our case [9]. A positive CSF culture establishes the diagnosis of fungal meningitis. However, in cases of failure to isolate a pathogen, perhaps because of the small inoculum size and slow growth of the yeast [8], a brain biopsy might be considered. Recommended appropriate therapy for CNS candidiasis is liposomal amphotericin B (3 to 5mg/kg) with or without 5-flucytosine 25mg/kg every 6 hours, for several weeks, followed by fluconazole 6 to 12mg/kg daily [8]. Despite the existing evidence for synergistic action between amphotericin and fluconazole, there is no confirmed clinical superiority of combination therapy for *Candida* yeast meningitis [10]. Despite appropriate combination treatment our patient died because of multiple brain lesions leading to diffuse brain edema. As autopsy was denied, confirmation of yeast involvement was achieved by histology and direct microscopic examination.

**Table 1:** Previously reported cohorts of central nervous system yeast infections

**Author and reference number**	**Cases (n)**	**Type of central nervous system infection (n)**	**Causative fungus**
Levy RM *et al*. [11]	5	Brain abscesses (4)	*Candida* species
*J Neurosurg. 1985 Apr;62 (4):475-95.*		Meningoencephalitis (1)	
Pappas PG *et al*. [6]	14	Meningitis (14)	*C. albicans* (13)
			*C. tropicalis* (1)
Dorko E *et al*. [12]	13	Meningitis (13)	*C. albicans (54%)*
*Folia Microbiol (Praha). 2002;47 (6):732-6.*			*C. parapsilosis (*23%)
			*C. tropicalis (15%)*
			*C. krusei (8%)*

## Conclusions

This is a report of a critically ill patient with an invasive CNS fungal infection in a tertiary hospital MSICU. Invasive fungal infections pose a difficult problem for the intensivist, owing both to the nature of the infection and the difficulty in diagnosis and treatment, and to the comorbidities of the critically ill. A multidisciplinary approach is frequently required, involving a combination of antifungal agents as well as surgical management where indicated. However, the mortality of invasive fungal infections in the ICU remains high in spite of efforts for prompt diagnosis and treatment.

## Consent

Written informed consent was obtained from the patient’s next of kin for publication of this case report and any accompanying images. A copy of the written consent is available for review by the Editor-in-Chief of this journal.

## Abbreviations

CNS: Central nervous system; CSF: Cerebrospinal fluid; CT: Computed tomography; ICU: Intensive care unit; MSICU: Medical-surgical intensive care unit; PCR: Polymerase chain reaction.

## Competing interests

The authors declare that they have no competing interests.

## Authors’ contributions

All authors read and approved the final manuscript and contributed to the design of the study.
